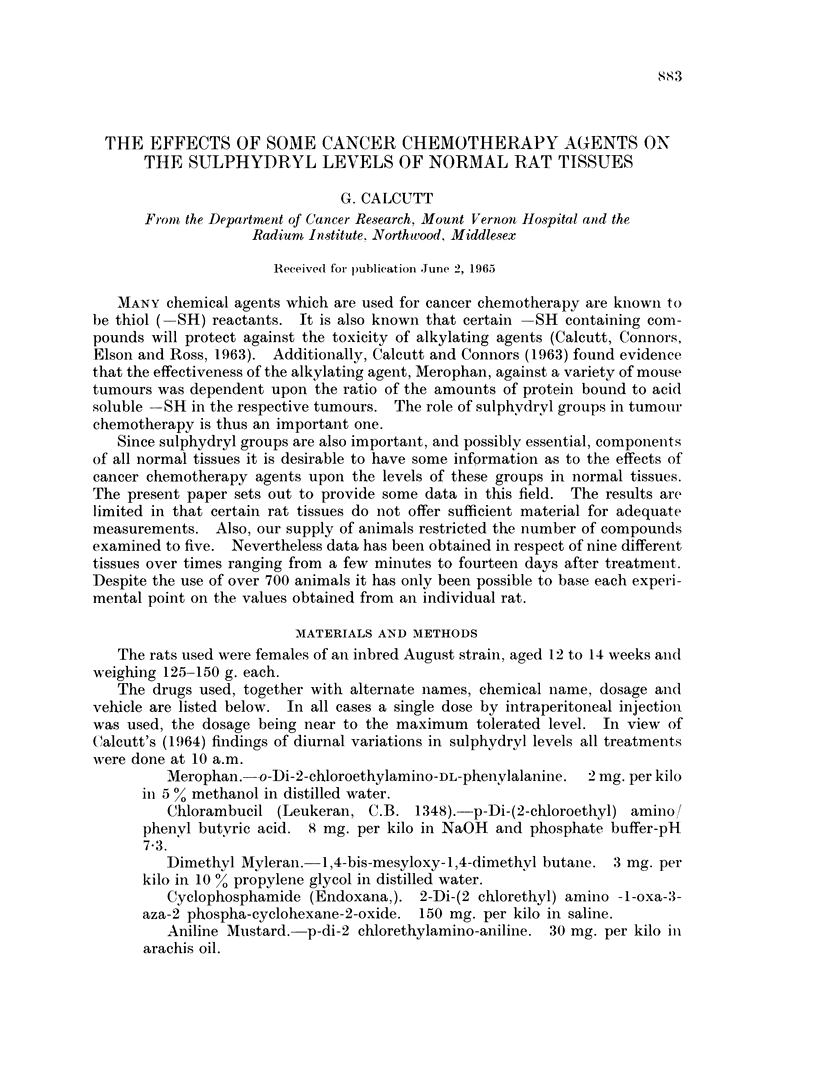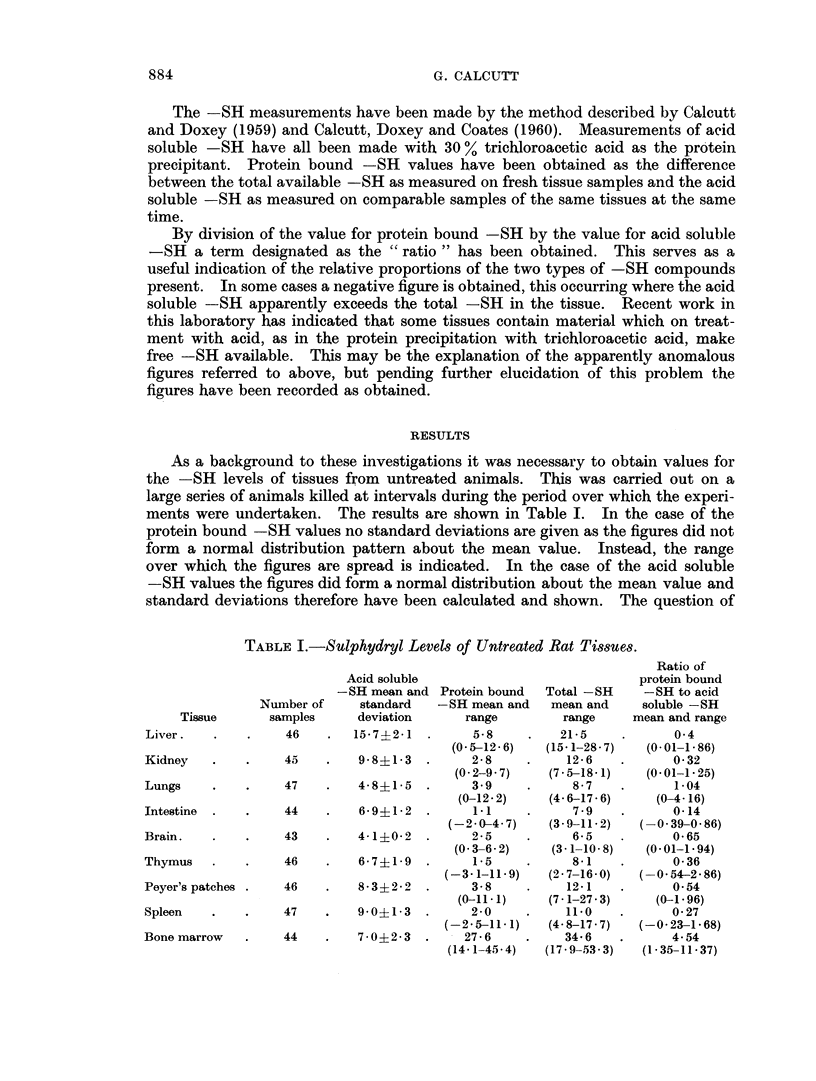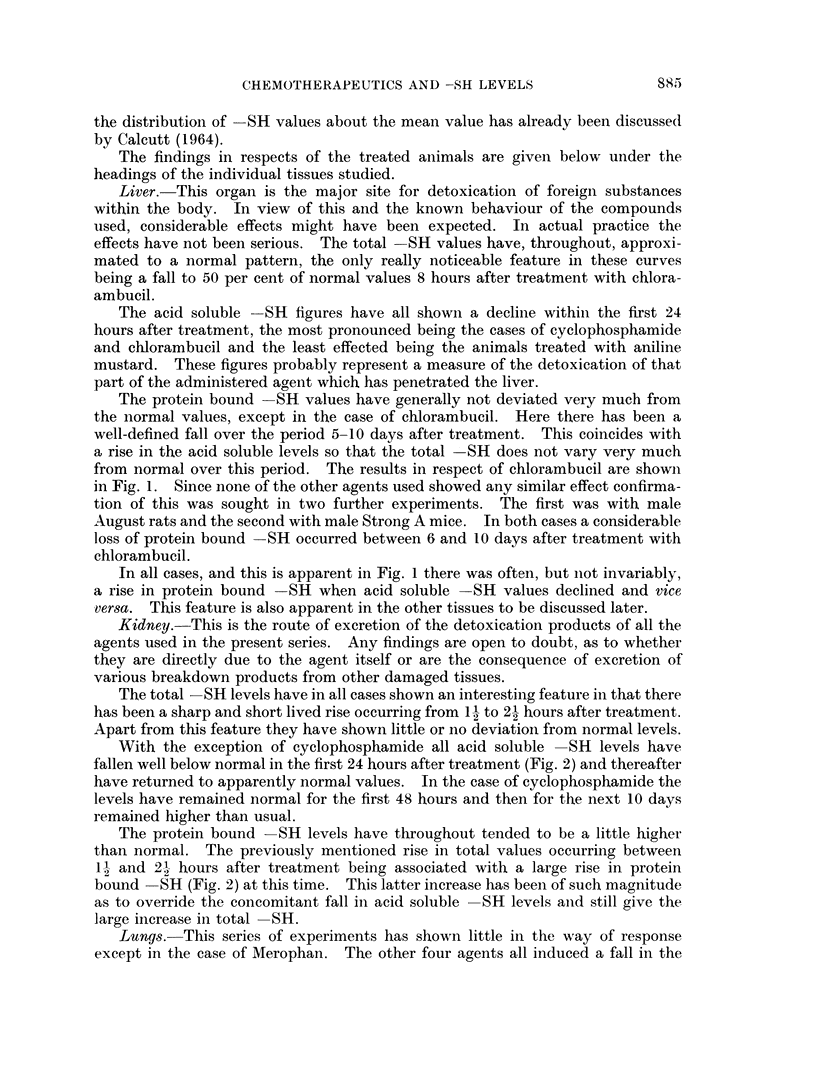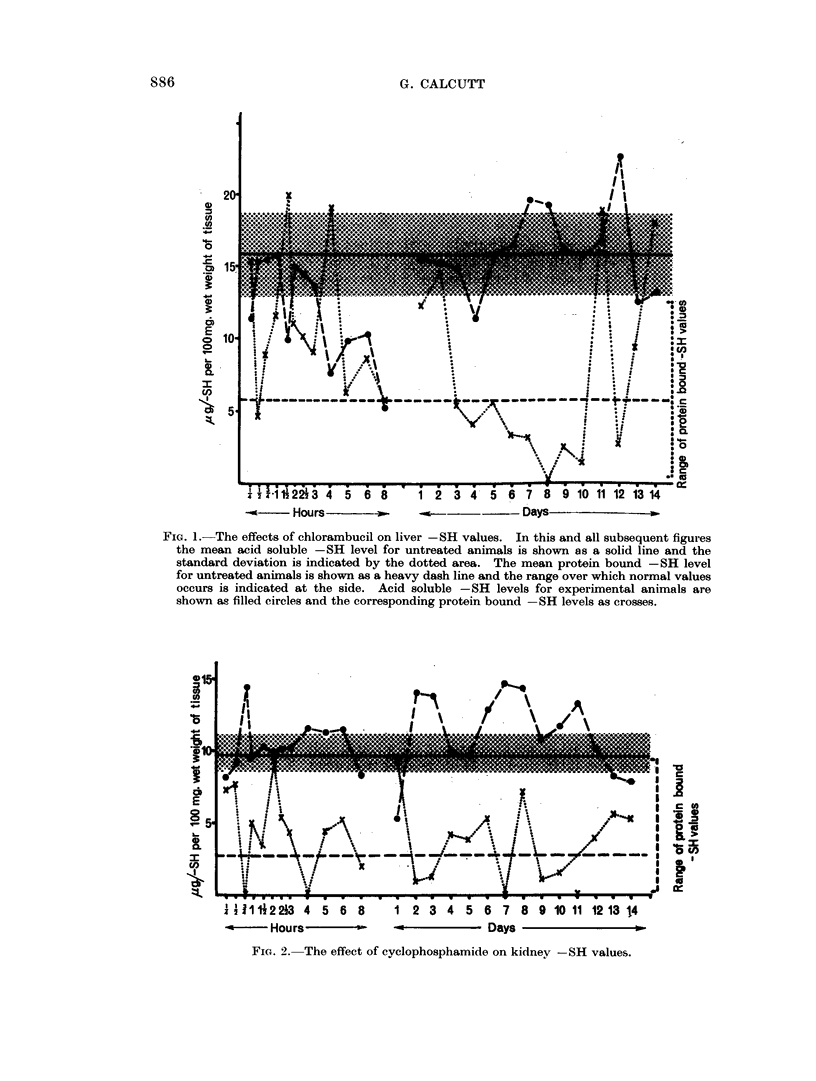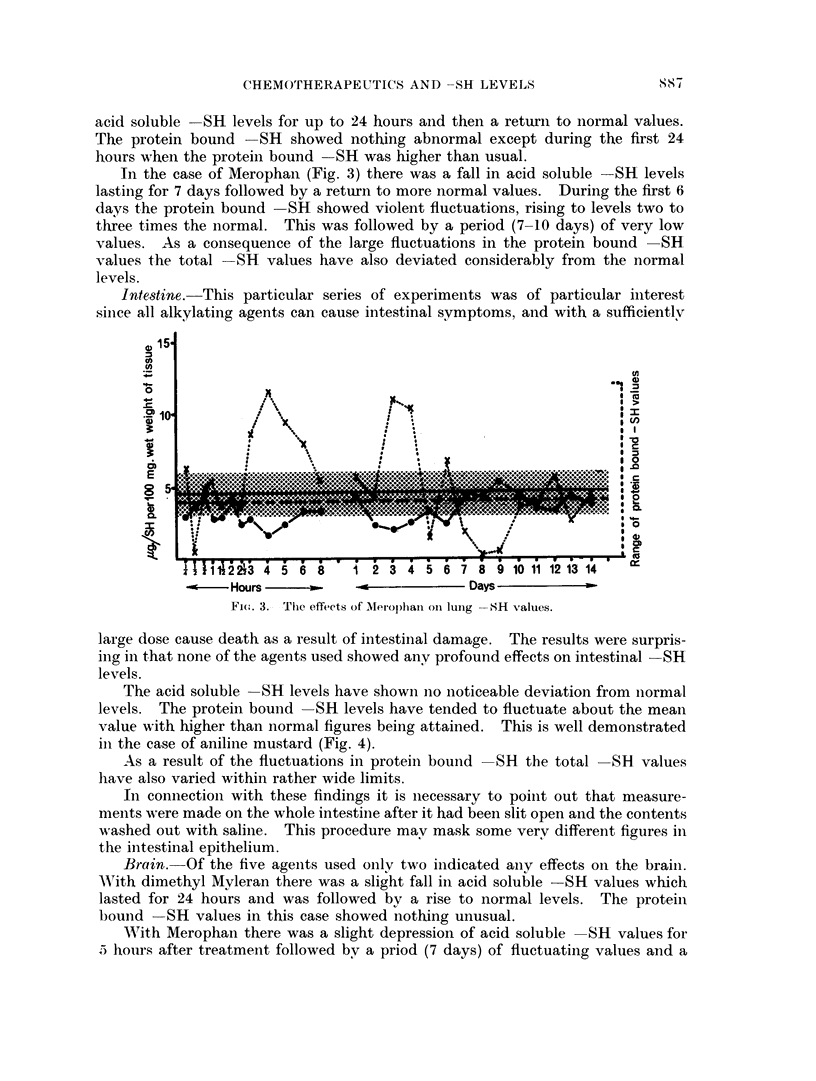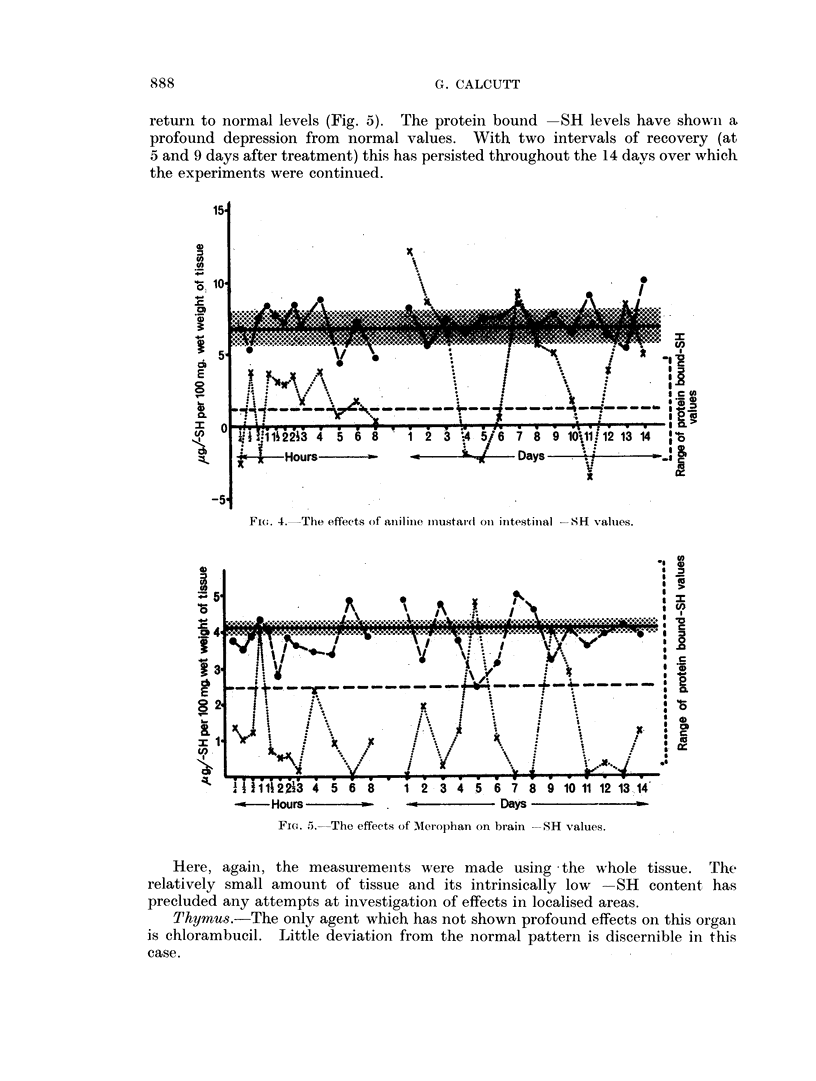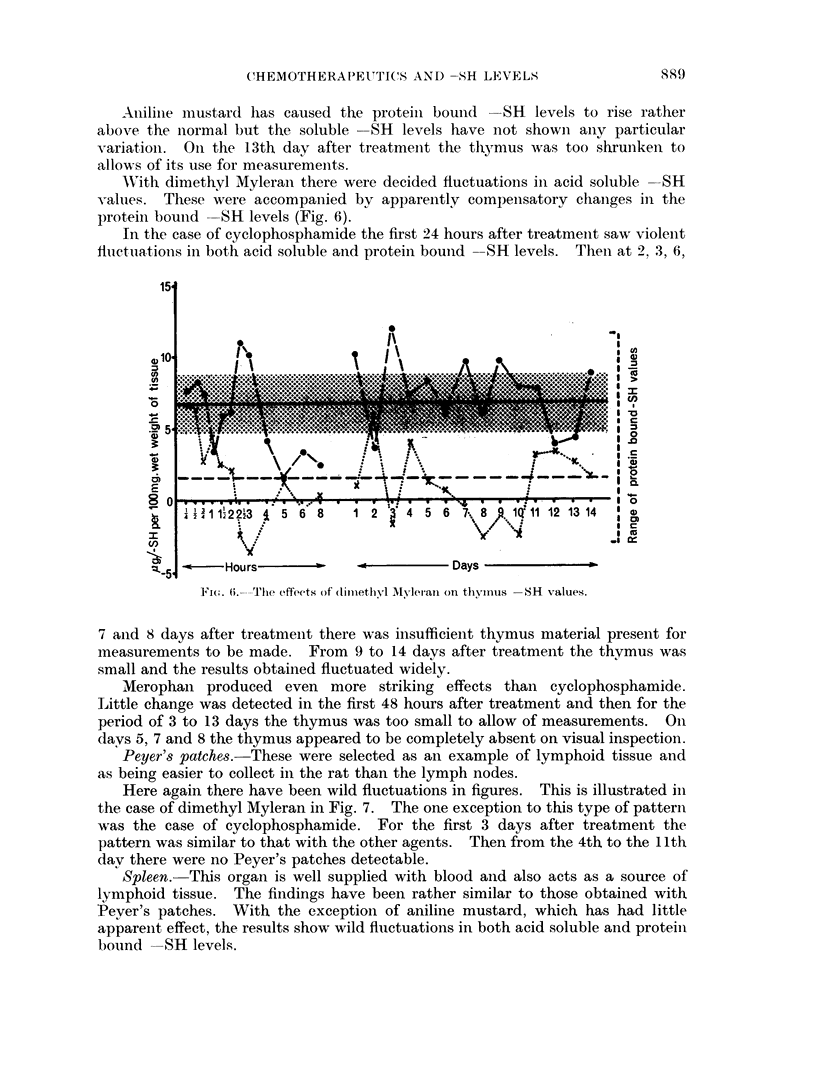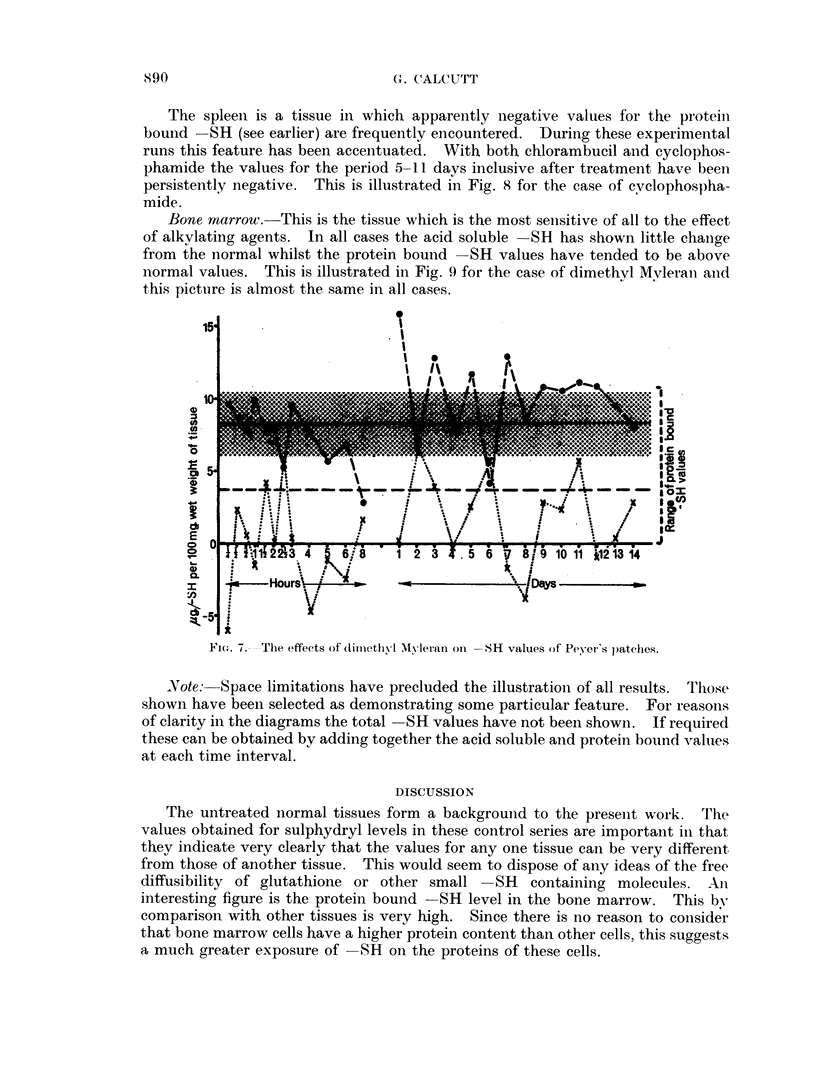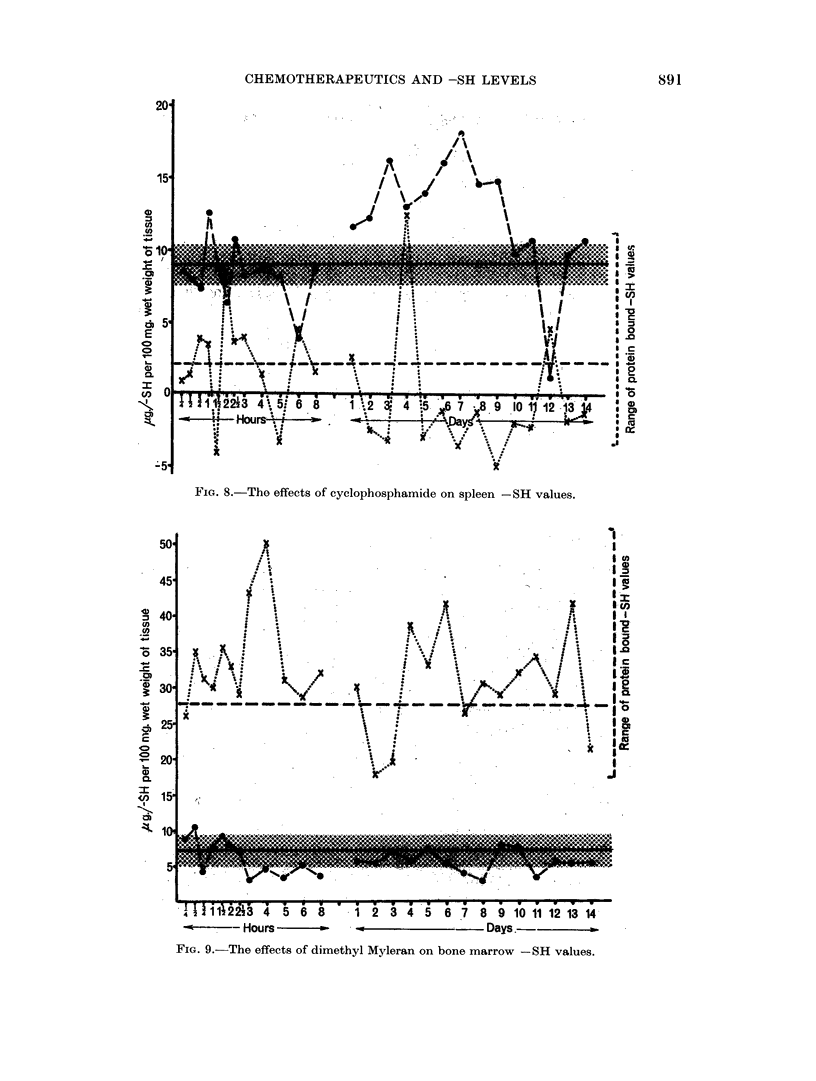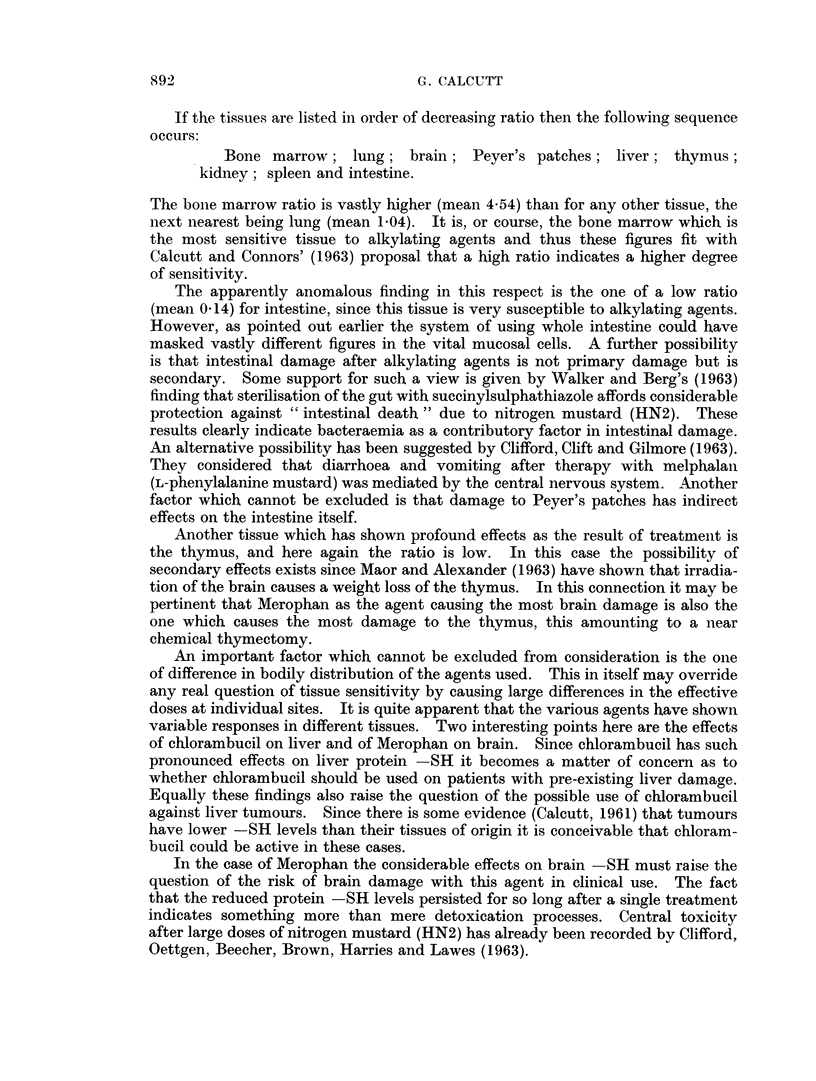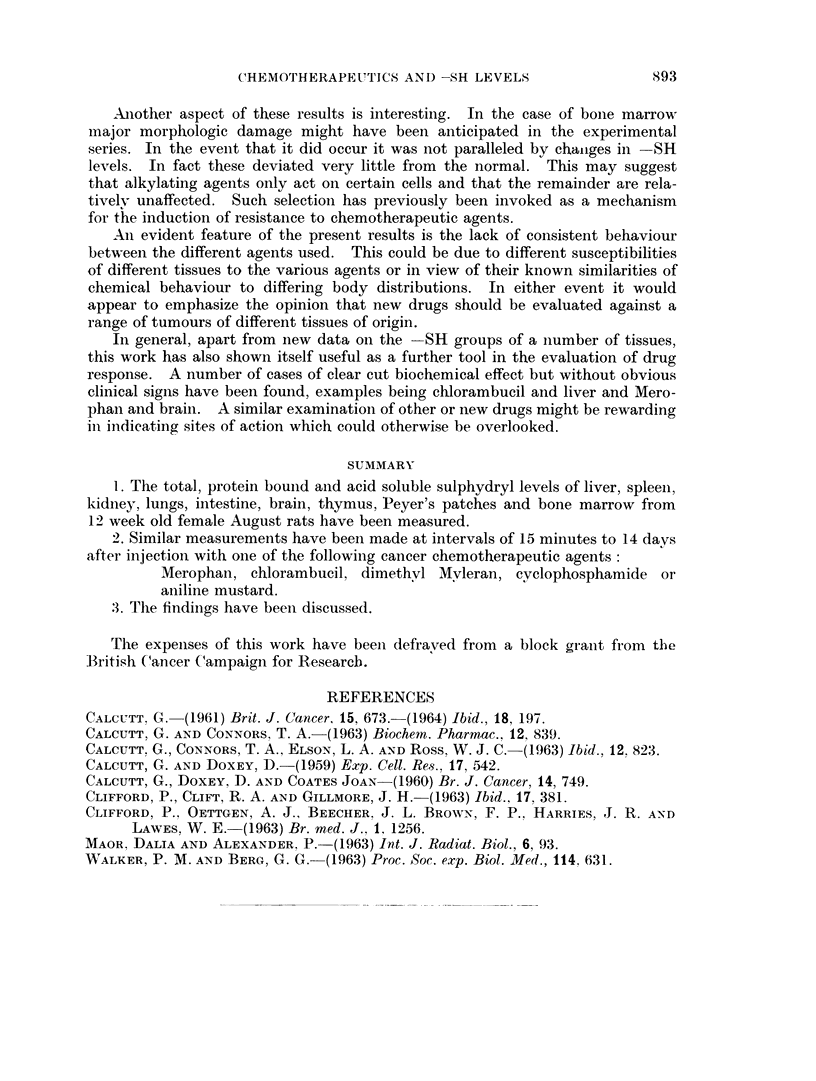# The effects of some cancer chemotherapy agents on the sulphydryl levels of normal rat tissues.

**DOI:** 10.1038/bjc.1965.103

**Published:** 1965-12

**Authors:** G. Calcutt


### THE EFFECTS OF SOME CANCER CHEMOTHERAPY AGENTS ON THE SULPHYDRYL LEVELS OF NORMAL RAT TISSUES

G. CALCUTT

*From the Department of Cancer Research, Mount Vernon Hospital and the Radium Institute, Northwood, Middlesex*

Received for publication June 2, 1965

MANY chemical agents which are used for cancer chemotherapy are known to be thiol (—SH) reactants. It is also known that certain —SH containing compounds will protect against the toxicity of alkylating agents (Calcutt, Connors, Elson and Ross, 1963). Additionally, Calcutt and Connors (1963) found evidence that the effectiveness of the alkylating agent, Merophan, against a variety of mouse tumours was dependent upon the ratio of the amounts of protein bound to acid soluble —SH in the respective tumours. The role of sulphydryl groups in tumour chemotherapy is thus an important one.

Since sulphydryl groups are also important, and possibly essential, components of all normal tissues it is desirable to have some information as to the effects of cancer chemotherapy agents upon the levels of these groups in normal tissues. The present paper sets out to provide some data in this field. The results are limited in that certain rat tissues do not offer sufficient material for adequate measurements. Also, our supply of animals restricted the number of compounds examined to five. Nevertheless data has been obtained in respect of nine different tissues over times ranging from a few minutes to fourteen days after treatment. Despite the use of over 700 animals it has only been possible to base each experimental point on the values obtained from an individual rat.

### MATERIALS AND METHODS

The rats used were females of an inbred August strain, aged 12 to 14 weeks and weighing 125–150 g. each.

The drugs used, together with alternate names, chemical name, dosage and vehicle are listed below. In all cases a single dose by intraperitoneal injection was used, the dosage being near to the maximum tolerated level. In view of Calcutt's (1964) findings of diurnal variations in sulphydryl levels all treatments were done at 10 a.m.

Merophan.—*o*-Di-2-chloroethylamino-DL-phenylalanine. 2 mg. per kilo in 5 % methanol in distilled water.

Chlorambucil (Leukeran, C.B. 1348).—p-Di-(2-chloroethyl) amino/phenyl butyric acid. 8 mg. per kilo in NaOH and phosphate buffer-pH 7·3.

Dimethyl Myleran.—1,4-bis-mesyloxy-1,4-dimethyl butane. 3 mg. per kilo in 10 % propylene glycol in distilled water.

Cyclophosphamide (Endoxana,). 2-Di-(2 chlorethyl) amino -1-oxa-3-aza-2 phospha-cyclohexane-2-oxide. 150 mg. per kilo in saline.

Aniline Mustard.—p-di-2 chlorethylamino-aniline. 30 mg. per kilo in arachis oil.

The —SH measurements have been made by the method described by Calcutt and Doxey (1959) and Calcutt, Doxey and Coates (1960). Measurements of acid soluble —SH have all been made with 30% trichloroacetic acid as the protein precipitant. Protein bound —SH values have been obtained as the difference between the total available —SH as measured on fresh tissue samples and the acid soluble —SH as measured on comparable samples of the same tissues at the same time.

By division of the value for protein bound —SH by the value for acid soluble —SH a term designated as the " ratio " has been obtained. This serves as a useful indication of the relative proportions of the two types of —SH compounds present. In some cases a negative figure is obtained, this occurring where the acid soluble —SH apparently exceeds the total —SH in the tissue. Recent work in this laboratory has indicated that some tissues contain material which on treatment with acid, as in the protein precipitation with trichloroacetic acid, make free —SH available. This may be the explanation of the apparently anomalous figures referred to above, but pending further elucidation of this problem the figures have been recorded as obtained.

### RESULTS

As a background to these investigations it was necessary to obtain values for the —SH levels of tissues from untreated animals. This was carried out on a large series of animals killed at intervals during the period over which the experiments were undertaken. The results are shown in Table I. In the case of the protein bound —SH values no standard deviations are given as the figures did not form a normal distribution pattern about the mean value. Instead, the range over which the figures are spread is indicated. In the case of the acid soluble —SH values the figures did form a normal distribution about the mean value and standard deviations therefore have been calculated and shown. The question of

TABLE I.—*Sulphydryl Levels of Untreated Rat Tissues.*

| Tissue | Number of samples | Acid soluble —SH mean and standard deviation | Protein bound —SH mean and range | Total —SH mean and range | Ratio of protein bound —SH to acid soluble —SH mean and range |
|---|---|---|---|---|---|
| Liver | 46 | 15·7±2·1 | 5·8 (0·5–12·6) | 21·5 (15·1–28·7) | 0·4 (0·01–1·86) |
| Kidney | 45 | 9·8±1·3 | 2·8 (0·2–9·7) | 12·6 (7·5–18·1) | 0·32 (0·01–1·25) |
| Lungs | 47 | 4·8±1·5 | 3·9 (0–12·2) | 8·7 (4·6–17·6) | 1·04 (0–4·16) |
| Intestine | 44 | 6·9±1·2 | 1·1 (−2·0–4·7) | 7·9 (3·9–11·2) | 0·14 (−0·39–0·86) |
| Brain | 43 | 4·1±0·2 | 2·5 (0·3–6·2) | 6·5 (3·1–10·8) | 0·65 (0·01–1·94) |
| Thymus | 46 | 6·7±1·9 | 1·5 (−3·1–11·9) | 8·1 (2·7–16·0) | 0·36 (−0·54–2·86) |
| Peyer's patches | 46 | 8·3±2·2 | 3·8 (0–11·1) | 12·1 (7·1–27·3) | 0·54 (0–1·96) |
| Spleen | 47 | 9·0±1·3 | 2·0 (−2·5–11·1) | 11·0 (4·8–17·7) | 0·27 (−0·23–1·68) |
| Bone marrow | 44 | 7·0±2·3 | 27·6 (14·1–45·4) | 34·6 (17·9–53·3) | 4·54 (1·35–11·37) |

the distribution of —SH values about the mean value has already been discussed by Calcutt (1964).

The findings in respects of the treated animals are given below under the headings of the individual tissues studied.

*Liver.*—This organ is the major site for detoxication of foreign substances within the body. In view of this and the known behaviour of the compounds used, considerable effects might have been expected. In actual practice the effects have not been serious. The total —SH values have, throughout, approximated to a normal pattern, the only really noticeable feature in these curves being a fall to 50 per cent of normal values 8 hours after treatment with chlorambucil.

The acid soluble —SH figures have all shown a decline within the first 24 hours after treatment, the most pronounced being the cases of cyclophosphamide and chlorambucil and the least effected being the animals treated with aniline mustard. These figures probably represent a measure of the detoxication of that part of the administered agent which has penetrated the liver.

The protein bound —SH values have generally not deviated very much from the normal values, except in the case of chlorambucil. Here there has been a well-defined fall over the period 5–10 days after treatment. This coincides with a rise in the acid soluble levels so that the total —SH does not vary very much from normal over this period. The results in respect of chlorambucil are shown in Fig. 1. Since none of the other agents used showed any similar effect confirmation of this was sought in two further experiments. The first was with male August rats and the second with male Strong A mice. In both cases a considerable loss of protein bound —SH occurred between 6 and 10 days after treatment with chlorambucil.

In all cases, and this is apparent in Fig. 1 there was often, but not invariably, a rise in protein bound —SH when acid soluble —SH values declined and *vice versa*. This feature is also apparent in the other tissues to be discussed later.

*Kidney.*—This is the route of excretion of the detoxication products of all the agents used in the present series. Any findings are open to doubt, as to whether they are directly due to the agent itself or are the consequence of excretion of various breakdown products from other damaged tissues.

The total —SH levels have in all cases shown an interesting feature in that there has been a sharp and short lived rise occurring from $1\frac{1}{2}$ to $2\frac{1}{2}$ hours after treatment. Apart from this feature they have shown little or no deviation from normal levels.

With the exception of cyclophosphamide all acid soluble —SH levels have fallen well below normal in the first 24 hours after treatment (Fig. 2) and thereafter have returned to apparently normal values. In the case of cyclophosphamide the levels have remained normal for the first 48 hours and then for the next 10 days remained higher than usual.

The protein bound —SH levels have throughout tended to be a little higher than normal. The previously mentioned rise in total values occurring between $1\frac{1}{2}$ and $2\frac{1}{2}$ hours after treatment being associated with a large rise in protein bound —SH (Fig. 2) at this time. This latter increase has been of such magnitude as to override the concomitant fall in acid soluble —SH levels and still give the large increase in total —SH.

*Lungs.*—This series of experiments has shown little in the way of response except in the case of Merophan. The other four agents all induced a fall in the

FIG. 1.—The effects of chlorambucil on liver —SH values. In this and all subsequent figures the mean acid soluble —SH level for untreated animals is shown as a solid line and the standard deviation is indicated by the dotted area. The mean protein bound —SH level for untreated animals is shown as a heavy dash line and the range over which normal values occurs is indicated at the side. Acid soluble —SH levels for experimental animals are shown as filled circles and the corresponding protein bound —SH levels as crosses.

FIG. 2.—The effect of cyclophosphamide on kidney —SH values.

acid soluble —SH levels for up to 24 hours and then a return to normal values. The protein bound —SH showed nothing abnormal except during the first 24 hours when the protein bound —SH was higher than usual.

In the case of Merophan (Fig. 3) there was a fall in acid soluble —SH levels lasting for 7 days followed by a return to more normal values. During the first 6 days the protein bound —SH showed violent fluctuations, rising to levels two to three times the normal. This was followed by a period (7–10 days) of very low values. As a consequence of the large fluctuations in the protein bound —SH values the total —SH values have also deviated considerably from the normal levels.

*Intestine.*—This particular series of experiments was of particular interest since all alkylating agents can cause intestinal symptoms, and with a sufficiently

FIG. 3. The effects of Merophan on lung —SH values.

large dose cause death as a result of intestinal damage. The results were surprising in that none of the agents used showed any profound effects on intestinal —SH levels.

The acid soluble —SH levels have shown no noticeable deviation from normal levels. The protein bound —SH levels have tended to fluctuate about the mean value with higher than normal figures being attained. This is well demonstrated in the case of aniline mustard (Fig. 4).

As a result of the fluctuations in protein bound —SH the total —SH values have also varied within rather wide limits.

In connection with these findings it is necessary to point out that measurements were made on the whole intestine after it had been slit open and the contents washed out with saline. This procedure may mask some very different figures in the intestinal epithelium.

*Brain.*—Of the five agents used only two indicated any effects on the brain. With dimethyl Myleran there was a slight fall in acid soluble —SH values which lasted for 24 hours and was followed by a rise to normal levels. The protein bound —SH values in this case showed nothing unusual.

With Merophan there was a slight depression of acid soluble —SH values for 5 hours after treatment followed by a priod (7 days) of fluctuating values and a return to normal levels (Fig. 5). The protein bound —SH levels have shown a profound depression from normal values. With two intervals of recovery (at 5 and 9 days after treatment) this has persisted throughout the 14 days over which the experiments were continued.

FIG. 4.—The effects of aniline mustard on intestinal —SH values.

FIG. 5.—The effects of Merophan on brain —SH values.

Here, again, the measurements were made using the whole tissue. The relatively small amount of tissue and its intrinsically low —SH content has precluded any attempts at investigation of effects in localised areas.

*Thymus*.—The only agent which has not shown profound effects on this organ is chlorambucil. Little deviation from the normal pattern is discernible in this case.

Aniline mustard has caused the protein bound —SH levels to rise rather above the normal but the soluble —SH levels have not shown any particular variation. On the 13th day after treatment the thymus was too shrunken to allows of its use for measurements.

With dimethyl Myleran there were decided fluctuations in acid soluble —SH values. These were accompanied by apparently compensatory changes in the protein bound —SH levels (Fig. 6).

In the case of cyclophosphamide the first 24 hours after treatment saw violent fluctuations in both acid soluble and protein bound —SH levels. Then at 2, 3, 6,

FIG. 6.—The effects of dimethyl Myleran on thymus —SH values.

7 and 8 days after treatment there was insufficient thymus material present for measurements to be made. From 9 to 14 days after treatment the thymus was small and the results obtained fluctuated widely.

Merophan produced even more striking effects than cyclophosphamide. Little change was detected in the first 48 hours after treatment and then for the period of 3 to 13 days the thymus was too small to allow of measurements. On days 5, 7 and 8 the thymus appeared to be completely absent on visual inspection.

*Peyer's patches.*—These were selected as an example of lymphoid tissue and as being easier to collect in the rat than the lymph nodes.

Here again there have been wild fluctuations in figures. This is illustrated in the case of dimethyl Myleran in Fig. 7. The one exception to this type of pattern was the case of cyclophosphamide. For the first 3 days after treatment the pattern was similar to that with the other agents. Then from the 4th to the 11th day there were no Peyer's patches detectable.

*Spleen.*—This organ is well supplied with blood and also acts as a source of lymphoid tissue. The findings have been rather similar to those obtained with Peyer's patches. With the exception of aniline mustard, which has had little apparent effect, the results show wild fluctuations in both acid soluble and protein bound —SH levels.

The spleen is a tissue in which apparently negative values for the protein bound —SH (see earlier) are frequently encountered. During these experimental runs this feature has been accentuated. With both chlorambucil and cyclophosphamide the values for the period 5–11 days inclusive after treatment have been persistently negative. This is illustrated in Fig. 8 for the case of cyclophosphamide.

*Bone marrow.*—This is the tissue which is the most sensitive of all to the effect of alkylating agents. In all cases the acid soluble —SH has shown little change from the normal whilst the protein bound —SH values have tended to be above normal values. This is illustrated in Fig. 9 for the case of dimethyl Myleran and this picture is almost the same in all cases.

FIG. 7.—The effects of dimethyl Myleran on —SH values of Peyer's patches.

*Note:*—Space limitations have precluded the illustration of all results. Those shown have been selected as demonstrating some particular feature. For reasons of clarity in the diagrams the total —SH values have not been shown. If required these can be obtained by adding together the acid soluble and protein bound values at each time interval.

### DISCUSSION

The untreated normal tissues form a background to the present work. The values obtained for sulphydryl levels in these control series are important in that they indicate very clearly that the values for any one tissue can be very different from those of another tissue. This would seem to dispose of any ideas of the free diffusibility of glutathione or other small —SH containing molecules. An interesting figure is the protein bound —SH level in the bone marrow. This by comparison with other tissues is very high. Since there is no reason to consider that bone marrow cells have a higher protein content than other cells, this suggests a much greater exposure of —SH on the proteins of these cells.

FIG. 8.—The effects of cyclophosphamide on spleen –SH values.

FIG. 9.—The effects of dimethyl Myleran on bone marrow –SH values.

If the tissues are listed in order of decreasing ratio then the following sequence occurs:

> Bone marrow ; lung ; brain ; Peyer's patches ; liver ; thymus ; kidney ; spleen and intestine.

The bone marrow ratio is vastly higher (mean 4·54) than for any other tissue, the next nearest being lung (mean 1·04). It is, or course, the bone marrow which is the most sensitive tissue to alkylating agents and thus these figures fit with Calcutt and Connors' (1963) proposal that a high ratio indicates a higher degree of sensitivity.

The apparently anomalous finding in this respect is the one of a low ratio (mean 0·14) for intestine, since this tissue is very susceptible to alkylating agents. However, as pointed out earlier the system of using whole intestine could have masked vastly different figures in the vital mucosal cells. A further possibility is that intestinal damage after alkylating agents is not primary damage but is secondary. Some support for such a view is given by Walker and Berg's (1963) finding that sterilisation of the gut with succinylsulphathiazole affords considerable protection against " intestinal death " due to nitrogen mustard (HN2). These results clearly indicate bacteraemia as a contributory factor in intestinal damage. An alternative possibility has been suggested by Clifford, Clift and Gilmore (1963). They considered that diarrhoea and vomiting after therapy with melphalan (L-phenylalanine mustard) was mediated by the central nervous system. Another factor which cannot be excluded is that damage to Peyer's patches has indirect effects on the intestine itself.

Another tissue which has shown profound effects as the result of treatment is the thymus, and here again the ratio is low. In this case the possibility of secondary effects exists since Maor and Alexander (1963) have shown that irradiation of the brain causes a weight loss of the thymus. In this connection it may be pertinent that Merophan as the agent causing the most brain damage is also the one which causes the most damage to the thymus, this amounting to a near chemical thymectomy.

An important factor which cannot be excluded from consideration is the one of difference in bodily distribution of the agents used. This in itself may override any real question of tissue sensitivity by causing large differences in the effective doses at individual sites. It is quite apparent that the various agents have shown variable responses in different tissues. Two interesting points here are the effects of chlorambucil on liver and of Merophan on brain. Since chlorambucil has such pronounced effects on liver protein —SH it becomes a matter of concern as to whether chlorambucil should be used on patients with pre-existing liver damage. Equally these findings also raise the question of the possible use of chlorambucil against liver tumours. Since there is some evidence (Calcutt, 1961) that tumours have lower —SH levels than their tissues of origin it is conceivable that chlorambucil could be active in these cases.

In the case of Merophan the considerable effects on brain —SH must raise the question of the risk of brain damage with this agent in clinical use. The fact that the reduced protein —SH levels persisted for so long after a single treatment indicates something more than mere detoxication processes. Central toxicity after large doses of nitrogen mustard (HN2) has already been recorded by Clifford, Oettgen, Beecher, Brown, Harries and Lawes (1963).

Another aspect of these results is interesting. In the case of bone marrow major morphologic damage might have been anticipated in the experimental series. In the event that it did occur it was not paralleled by changes in —SH levels. In fact these deviated very little from the normal. This may suggest that alkylating agents only act on certain cells and that the remainder are relatively unaffected. Such selection has previously been invoked as a mechanism for the induction of resistance to chemotherapeutic agents.

An evident feature of the present results is the lack of consistent behaviour between the different agents used. This could be due to different susceptibilities of different tissues to the various agents or in view of their known similarities of chemical behaviour to differing body distributions. In either event it would appear to emphasize the opinion that new drugs should be evaluated against a range of tumours of different tissues of origin.

In general, apart from new data on the —SH groups of a number of tissues, this work has also shown itself useful as a further tool in the evaluation of drug response. A number of cases of clear cut biochemical effect but without obvious clinical signs have been found, examples being chlorambucil and liver and Merophan and brain. A similar examination of other or new drugs might be rewarding in indicating sites of action which could otherwise be overlooked.

### SUMMARY

1. The total, protein bound and acid soluble sulphydryl levels of liver, spleen, kidney, lungs, intestine, brain, thymus, Peyer's patches and bone marrow from 12 week old female August rats have been measured.

2. Similar measurements have been made at intervals of 15 minutes to 14 days after injection with one of the following cancer chemotherapeutic agents :

Merophan, chlorambucil, dimethyl Myleran, cyclophosphamide or aniline mustard.

3. The findings have been discussed.

The expenses of this work have been defrayed from a block grant from the British Cancer Campaign for Research.

### REFERENCES

CALCUTT, G.—(1961) *Brit. J. Cancer*, **15**, 673.—(1964) *Ibid.*, **18**, 197.

CALCUTT, G. AND CONNORS, T. A.—(1963) *Biochem. Pharmac.*, **12**, 839.

CALCUTT, G., CONNORS, T. A., ELSON, L. A. AND ROSS, W. J. C.—(1963) *Ibid.*, **12**, 823.

CALCUTT, G. AND DOXEY, D.—(1959) *Exp. Cell. Res.*, **17**, 542.

CALCUTT, G., DOXEY, D. AND COATES JOAN—(1960) *Br. J. Cancer*, **14**, 749.

CLIFFORD, P., CLIFT, R. A. AND GILLMORE, J. H.—(1963) *Ibid.*, **17**, 381.

CLIFFORD, P., OETTGEN, A. J., BEECHER, J. L. BROWN, F. P., HARRIES, J. R. AND LAWES, W. E.—(1963) *Br. med. J.*, **1**, 1256.

MAOR, DALIA AND ALEXANDER, P.—(1963) *Int. J. Radiat. Biol.*, **6**, 93.

WALKER, P. M. AND BERG, G. G.—(1963) *Proc. Soc. exp. Biol. Med.*, **114**, 631.